# Utilization of Biopolymer-Based Lutein Emulsion as an Effective Delivery System to Improve Lutein Bioavailability in Neonatal Rats

**DOI:** 10.3390/nu16030422

**Published:** 2024-01-31

**Authors:** Yanqi Zhang, Lingyan Kong, Jeannine C. Lawrence, Libo Tan

**Affiliations:** Department of Human Nutrition and Hospitality Management, University of Alabama, Tuscaloosa, AL 35487, USA; yzhang309@crimson.ua.edu (Y.Z.); lkong@ches.ua.edu (L.K.); jlawrence@ches.ua.edu (J.C.L.)

**Keywords:** lutein, biopolymer, oil-in-water emulsion, bioavailability, neonatal health

## Abstract

Newborns’ eyes and brains are prone to oxidative stress. Lutein has antioxidant properties and is the main component of macular pigment essential for protecting the retina, but has low bioavailability, thereby limiting its potential as a nutritional supplement. Oil-in-water emulsions have been used as lutein delivery systems. In particular, octenylsuccinated (OS) starch is a biopolymer-derived emulsifier safe to use in infant foods, while exhibiting superior emulsifying capacity. This study determined the effects of an OS starch-stabilized lutein emulsion on lutein bioavailability in Sprague-Dawley neonatal rats. In an acute study, 10-day-old pups received a single oral dose of free lutein or lutein emulsion, with subsequent blood sampling over 24 h to analyze pharmacokinetics. The lutein emulsion group had a 2.12- and 1.91-fold higher maximum serum lutein concentration and area under the curve, respectively, compared to the free lutein group. In two daily dosing studies, oral lutein was given from postnatal day 5 to 18. Blood and tissue lutein concentrations were measured. The results indicated that the daily intake of lutein emulsion led to a higher lutein concentration in circulation and key tissues compared to free lutein. The OS starch-stabilized emulsion could be an effective and safe lutein delivery system for newborns.

## 1. Introduction

Optimizing early-life health and development are crucial for lifelong wellness. During infancy, a particularly vulnerable period in the life-span, newborns are exposed to elevated oxidative stress and free radical toxicity due to their high susceptibility to environmental perturbation [1]. Oxidative stress results from an imbalance between antioxidants and the production of free radicals. At birth, infants transition from the hypoxic uterine environment, i.e., 20–25 mmHg PO_2_, to a relatively hyperoxic environment, i.e., 100 mmHg PO_2_. Such elevated oxygen levels may cause oxidative stress and escalate free radical production, potentially damaging cells and tissues. This harm can lead to conditions such as respiratory distress syndrome, bronchopulmonary dysplasia, brain injury, and retinopathy of prematurity (ROP), known collectively as “oxygen radical disease of neonatology” [2]. Additionally, the dynamic immune changes of infants and reduced proinflammatory responses in the first months increase their susceptibility to infection [3,4]. Recent studies link pro-inflammatory cytokines to various newborn diseases, including pulmonary issues, ROP, perinatal brain injury, and immune dysfunction [5,6,7]. Thus, optimizing newborn and infant nutrition, particularly through bioactive compounds with antioxidant and anti-inflammatory properties, has emerged as a key strategy for promoting their health and development.

Lutein is a hydrophobic xanthophyll carotenoid. It is found abundantly in green leafy vegetables, yellow-orange fruits and vegetables, marigold flowers, and egg yoks in a free or esterified form [8,9]. As humans cannot synthesize lutein, it must be obtained through diet. In the body, lutein has antioxidant properties due to its conjugated double bonds [10], and is concentrated in the retina’s macular region. It forms the main component of macular pigment, filtering blue light and protecting against oxidative damage [11]. Lutein and its isomer zeaxanthin are the primary carotenoids in human milk [12]. Newborns, with their retinas being more permeable to blue light than adults, particularly benefit from lutein for visual health [13]. Lutein also possesses anti-inflammatory effects and is the most abundant carotenoid in the infant brain [14,15]. It is worth noting that certain at-risk infant groups have compromised lutein status. Preterm infants, who are highly susceptible to oxidative stress and related newborn diseases, were reported to have significantly lower serum and brain lutein concentrations than full-term infants [16,17]. Additionally, formula-fed infants were found to have lower serum lutein concentrations than breastfed infants [18,19]. A previous study reported that lutein/zeaxanthin concentration increased from 48 µg/L at birth to 96 µg/L at one month in breastfed newborns, while it decreased from 49 µg/L to 33 µg/L in infants fed with a formula that was not fortified with lutein [19]. Therefore, increasing lutein intake through supplementation or fortification could benefit infants, especially those at risk.

However, the potential of lutein as a nutritional intervention is hindered by its low stability, poor water solubility, and low oral bioavailability [20]. As a lipophilic compound, lutein depends on the formation of micelles with lipids and bile acids in the small intestine for absorption, resulting in a lower absorption rate as compared to water-soluble substances [21]. Lutein in supplements is generally in the esterified form, as it is extracted from marigold flowers [8,9]. Lutein ester is known to be even less bioavailable than the free form because esterified lutein requires an additional ester hydrolysis step in the small intestine [9,22]. The susceptibility of lutein to degradation by heat, light, and oxygen further compromises its potential as a nutritional supplement or fortification. It has been shown that compared to breast-fed infants, infants consuming lutein-fortified formula needed to consume four-fold higher lutein dose to possess the same serum lutein level as of breast-fed infants [18].

Encapsulation systems, such as oil-in-water (O/W) emulsion, have shown promise in enhancing lutein stability and bioavailability in adult human and rodent models [20,23]. An O/W emulsion contains oil droplets carrying the lipophilic compound and dispersed in a continuous water phase, stabilized by emulsifiers. The emulsion system may enhance the absorption of a bioactive compound through reducing droplet size, protecting the compound from degradation in the acidic stomach environment, assisting in its incorporation into mixed micelles, and enhancing its intestinal permeability [24,25,26]. When developing infant foods, it is important to carefully choose emulsifiers, as some synthetic emulsifiers may raise health-related concerns [27,28]. Recent studies have explored the use of emulsifiers derived from food-grade biopolymers, such as proteins and polysaccharides, including octenylsuccinated (OS) starch [29,30,31].

The current study uses octenylsuccinated (OS) starch as the emulsifier in the lutein emulsion. OS starches are cost-effective, commercially available biopolymers that have been approved to be used in infant foods at the maximum level of 20,000 mg/kg per day [32]. They are derived from natural sources and are commonly used as emulsifiers in food and beverage products [33]. In our latest research, we reported the effectiveness of 3 types of OS starch on the droplet aggregation, storage stability, and in vitro bioaccessibility of lutein [34]. Of the 3 emulsifiers, Capsule TA (CTA)-stabilized lutein emulsion exhibited the overall best performance in droplet size, physical stability, chemical stability, and in vitro release; therefore, it was selected for the current in vivo research [34]. The aim of the present study was to determine if the CTA-stabilized lutein emulsion could improve the lutein bioavailability and tissue lutein status, especially in the eye and the brain, in neonatal Sprague-Dawley rats.

## 2. Materials and Methods

### 2.1. Chemicals and Materials

Lutein ester (Xangold^®^ 30% OLV) was generously provided by BASF Inc. (Florham Park, NJ, USA). This sample contains 284.0 mg/g of mixed lutein esters, mainly lutein dipalmitate, and 14.5 mg/g of zeaxanthin esters isolated from marigold flowers (*Tagetes erecta*). Extra virgin olive oil (Badia Spices, Doral, FL, USA) was purchased from a local grocery store. OS starch (CTA) was kindly provided by Ingredion (Westchester, IL, USA). Tetrahydrofuran (THF) and other reagents of analytical grade were purchased from VWR International Inc. (Radnor, PA, USA).

### 2.2. Preparation of CTA-Based Lutein Emulsion

The organic phase was prepared by dissolving lutein ester in olive oil. The aqueous phase was prepared by adding 3 g of CTA into 10 mL of deionized water and stirring overnight until fully dispersed. The CTA-stabilized lutein emulsion was obtained by mixing the biopolymer dispersion and lutein oil at a ratio of 30:70 (i.e., 70% oil volume fraction) using a mechanical homogenizer (VWR 200, Radnor, PA, USA) at 20,000 rpm for 2 min [31].

#### 2.2.1. Microstructure

The microstructure of CTA- stabilized lutein emulsion was observed using confocal laser scanning microscope (CLSM). Specifically, 1 mL of lutein emulsion was combined with 10 μL of Nile blue staining solution (1 mg/mL) and thoroughly mixed for 30 to 60 s using a vortex mixer, ensuring even distribution of the dye throughout the emulsion. Then, the mixture was incubated for one hour in a dark room. For microscopic examination, a drop of the dye-stained lutein emulsion was carefully applied to a microscope slide and covered with a cover slip. The dye-stained emulsion was then observed using a CLSM (Nikon C2, Melville, NY, USA) equipped with a LUN4 4 Line Solid State Laser System (Nikon, Melville, NY, USA). The 60× oil immerse objective lenses were used, and the excitation wavelength was set at 488 nm for lutein and 640 nm for Nile blue. Images from two representative regions of the emulsion sample were captured and were measured for number-weighted mean droplet size using Image J2 2.3.0 software (Bethesda, MD, USA).

#### 2.2.2. Encapsulation Efficiency

Encapsulation efficiency is the amount of lutein completely encapsulated in the O/W emulsion instead of floating on the surface of the emulsion. Lutein content in the CTA-stabilized lutein emulsion was determined through solvent extraction and spectrophotometry. A quantity of 0.1 g of emulsion sample was combined with 4.99 mL of THF and subjected to disruption using a homogenizer (VWR 200, Radnor, PA, USA) at a speed of 10,000 rpm for a duration of 30 s. The resultant mixture underwent centrifugation at 3000× *g* for 15 min, and subsequently, the supernatant was gathered. The lutein concentration within the supernatant was determined by measuring absorbance at 452 nm using U*V*/*v*is spectrophotometry (Mettler Toledo, OH, USA) [35]. A standard calibration curve for lutein in THF was generated using Xangold^®^ 30% OLV. The calibration curve (range of 0.05 to 0.003125 μg/mL) displayed remarkable linear correlation with a strong coefficient (r^2^ = 0.9997). The encapsulation efficiency was computed using a formula adapted from a previous study [35]:

$$\mathrm{Encapsulation}\;\mathrm{efficiency}\;(\%)=\frac{Amount\;of\;lutein\;detected\;by\;UV-vis}{Amount\;of\;lutein\;loaded\;in\;the\;emulsion}\times 100\%$$

### 2.3. Acute Dosing Study—Pharmacokinetic Parameters and Bioavailability of Lutein

#### 2.3.1. Animal Experiment

**Figure 1:**
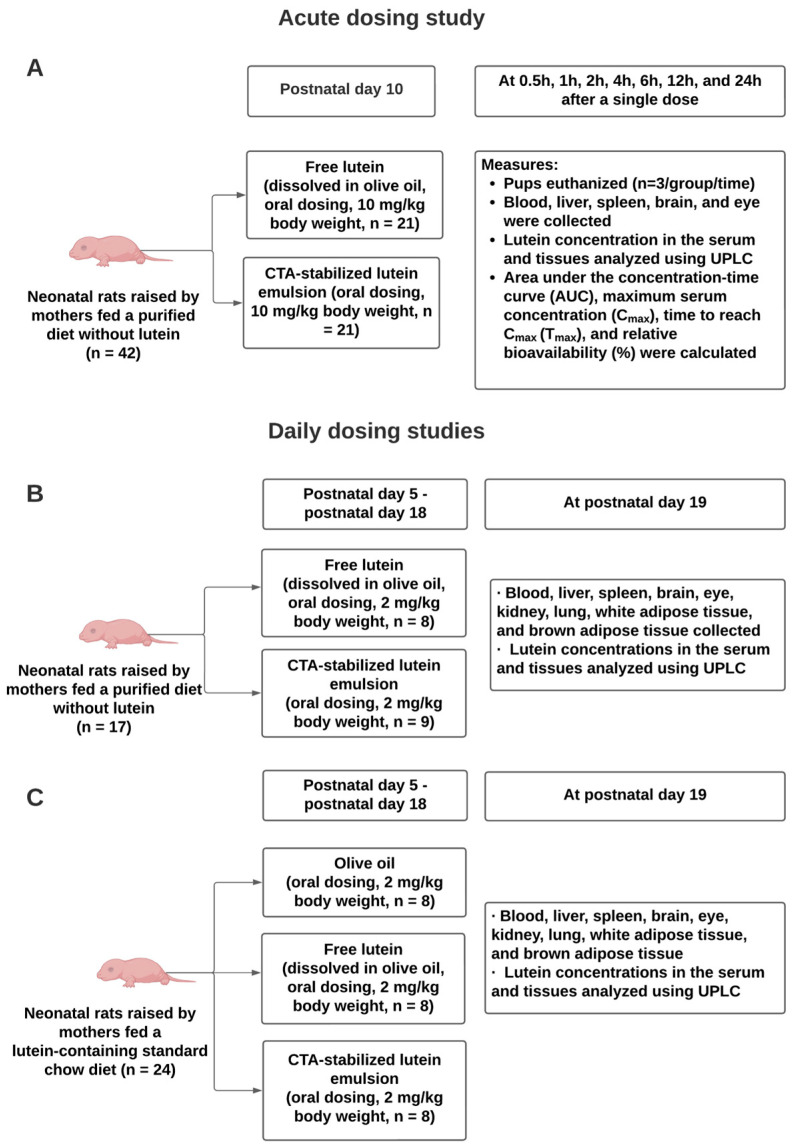
Schematic diagrams of the study design of the acute dosing study (**A**) and daily dosing studies, in which maternal rats consumed a purified diet without lutein (**B**) and a lutein-containing standard chow diet (**C**), respectively.

To determine the bioavailability of lutein in neonatal Sprague-Dawley rats, an acute dosing study was conducted. The procedure was approved by the Institutional Animal Care and Use Committee of the University of Alabama (Number: 21-08-4864, 23 September 2021). The schematic diagram of the study design is shown in Figure 1A. On postnatal day 10 (P10), neonatal rats were randomly selected from litters and received a single dose of either free lutein (i.e., lutein ester dissolved in olive oil) or lutein emulsion, both containing 10 mg/kg body weight of lutein. The dose was administered to the pup’s mouth via a pipette. The concentration of lutein ester in the oil phase was 0.004 g/mL. The lutein dosage was selected based on our pilot study and previous studies [36,37]. It is higher than the dosage used in human newborns but is considered acceptable and safe to be used in pharmacokinetic studies in rodent models [20]. At 0.5 h, 1 h, 2 h, 4 h, 6 h, 12 h, and 24 h after dosing, pups (*n* = 3/time point/group) were euthanized, and blood, liver, spleen, brain, and eye were collected. Serum was obtained by centrifuging blood samples and tissues were stored at −80 °C until time of analysis. A group of rat pups (*n* = 3) not receiving treatments were used to measure the baseline lutein concentrations. Throughout the study, maternal rats consumed a purified diet with no lutein (D19120501, Research Diets Inc, New Brunswick, NJ, USA) (Table 1). 

#### 2.3.2. Analysis of Lutein Concentration in Serum, Liver, Spleen, Brain, and Eye

Serum and tissue lutein concentration was determined using an ultra-performance liquid chromatography (UPLC) system (Waters, Milford, MA, USA) equipped with a photo-diode array detector (PDA)/MS/MS, according to our well-established methods with slight modification [38]. As for the serum, 100 μL of the sample was incubated with 2 mL of 100% ethanol at room temperature for an hour for lipid extraction. For the tissues, ~0.1 g of the sample was weighed, homogenized, and incubated with 2 mL of 100% ethanol at room temperature for an hour. An amount of 1 mL of 5% (*w*/*v*) potassium hydroxide was added to the sample and incubated in a water bath at 60 °C for 45 min for saponification. Upon saponification, samples were allowed to cool for about 15 min, and then an addition of 4 mL of hexane containing 0.1% butylated hydroxytoluene (*w*/*v*) and 2 mL of deionized water was added. The mixture was vortexed for 20 s and centrifuged at 1600 rpm at 25 °C for 15 min. The upper layer was collected following centrifugation and dried under nitrogen at 37 °C. The dried sample was then rinsed twice with hexane, dried, and reconstituted with 100 µL of acetonitrile-methanol. The reconstituted sample was centrifuged for 2 min to remove any possible precipitation. For UPLC analysis, 10 µL of the sample was injected into the Waters Acquity UPLC HSS T3 column. Solvent A was acetonitrile: methanol (50:50, *v*/*v*), and solvent B was 100% isopropanol. The mobile phase was made up of solvent A and solvent B in a volume ratio of 95:5 (*v*/*v*). The flow rate was set as 0.5 mL/min, and the column temperature was maintained at 35 °C. The detection wavelength was set at 446 nm. Retinyl acetate (Sigma-Aldrich, St. Louis, MO, USA) was used as the internal standard [39].

#### 2.3.3. Bioavailability Analysis

Bioavailability is defined as the rate and range of the active compound to the systemic circulation through a single oral administration [40,41]. It can be evaluated by pharmacokinetic parameters derived from the compound kinetic curve (serum concentration vs. time). In the acute dosing study, pharmacokinetic parameters, including maximum lutein concentration (C_max_), time to reach C_max_ (T_max_), and area under the time-concentration curve (AUC_0–24_) were determined. C_max_ and T_max_ were read directly from the serum lutein kinetic curve. The area under the time-concentration curve (AUC_0–24_) was calculated by trapezoid rule with linear interpolation using GraphPad Prism 8.0 (San Diego, CA, USA) [42]. The relative bioavailability of lutein emulsion to free lutein was calculated using the following equation [37]:

$$\mathrm{Relative}\;\mathrm{bioavailability}\;(\%)=\frac{AUCexperiment}{AUCcontrol}\times 100$$

### 2.4. Daily Dosing Studies in Neonatal Rats

#### 2.4.1. Animal Experiments

To determine the effects of daily consumption of CTA-stabilized lutein emulsion on tissue lutein status, two daily dosing studies were conducted. The procedures were approved by the Institutional Animal Care and Use Committee of the University of Alabama (Number: 21-02-4373, 4 February 2021). The schematic diagram is shown in Figure 1B,C. For daily dosing study 1 (Figure 1B), on P5, rat pups (*n* = 8–9/group) were randomly selected from the litters and received an oral dose of either free lutein (i.e., lutein ester dissolved in olive oil) or lutein emulsion both containing 2 mg/kg body weight of lutein. Our previous data indicated that when maternal rats were fed a diet devoid of lutein, lutein was not detected in the offspring; therefore, a negative control group was not included. Dosing was repeated daily until P18. The dosage was determined from previous research in both rodent and human newborns and was also established as an Acceptable Daily Intake for lutein by the Joint Food and Agricultural Organization/World Health Organization [43,44,45,46]. On P19, pups were euthanized. Rats are known to wean between P19 and P21 [47]. Blood from vena cava, liver, spleen, brain, eye, kidney, lung, white adipose tissue (WAT), and brown adipose tissue (BAT) were collected. Blood samples were centrifuged to obtain serum. Serum and collected tissues were frozen at −80 °C until analysis.

To mimic the human dietary intake of lutein, daily dosing study 2 was conducted (Figure 1C). In this study, maternal rats consumed a standard rodent chow diet containing lutein from ingredients (i.e., ground corn) during pregnancy and lactation (Figure S2). The study design was similar to that of daily dosing study 1, except that a negative control group, in which rat pups were given olive oil from P5 to P18, was included in this study.

#### 2.4.2. Analysis of Lutein Concentration in Serum and Tissues

Serum and tissue lutein concentrations were analyzed using the methods described in Section 2.3.2.

### 2.5. Statistical Analysis

Data are reported as mean ± standard deviation (SD). Data is normally distributed. Differences between two groups were determined using Student’s *t*-test, while those among groups of more than two were assessed using one-way ANOVA followed by a Bonferroni posttest. Statistical significance was set as *P* < 0.05. Data were analyzed in GraphPad Prism 8.0 (San Diego, CA, USA).

## 3. Results

### 3.1. Microstructure of Lutein Emulsion Stabilized by 30% (w/v) CTA

**Figure 2:**
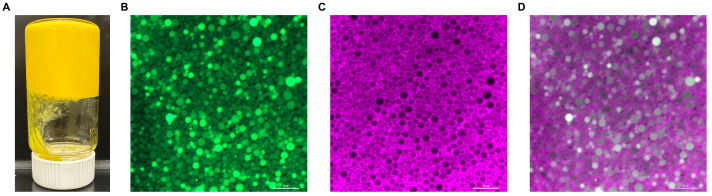
Photo (**A**) and the confocal laser scanning microscopy images of the oil phase florescence filed (**B**), aqueous phase fluorescence field (**C**), and overlap fluorescence field (**D**) of the lutein emulsion stabilized by 30% *w*/*v* capsule TA^®^ (CTA). Lutein in oil phase was naturally fluorescent and was excited at 488 nm in green; CTA was stained with Nile blue and excited at 633 nm in purple; bar is 10 μm, using 60× oil immerse objective lens.

The photo and the micrographs of CTA-stabilized lutein emulsion were shown in Figure 2. CTA-stabilized lutein emulsion had no phase separation and was self-standing and immobile after putting the bottle upside down, indicating the formation of “emulsion gel” [31]. The green fluorescence field (Figure 2B), purple fluorescence field (Figure 2C), and overlap fluorescence filed (Figure 2D) were shown in the CLSM images of CTA-stabilized lutein emulsion, which correspond to exciting natural lutein fluorescence, Nile blue staining, and both, respectively. As can be seen, the lutein oil droplets were homogenized and close-packed. The aqueous phase was located at the surface of the droplets and encapsulated the oil droplets in between, indicating the successful formation of the O/W emulsion.

The mean droplet diameter, minimum droplet diameter, and maximum droplet diameter of the lutein emulsion was 1.73 ± 1.14 μm, 0.08 μm, and 4.99 μm, respectively (Table 2). The encapsulation efficiency was 0.89 ± 0.014%.

### 3.2. Acute Dosing Study—Serum Lutein Kinetics

**Figure 3:**
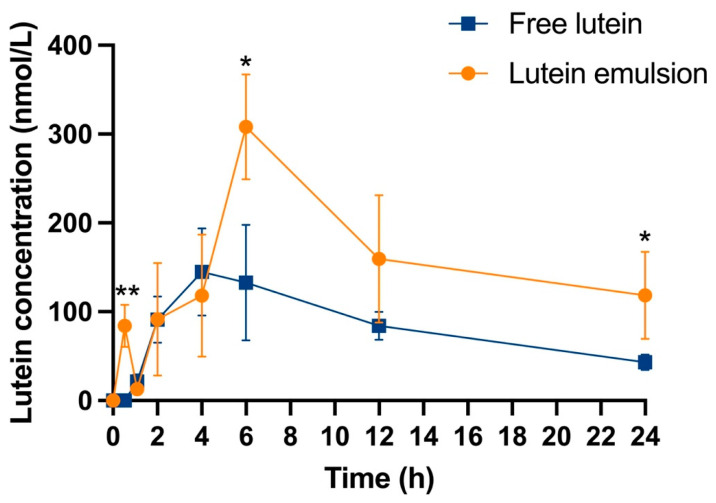
Serum concentration-time curve of lutein of rat pups after either a single dose of free lutein or CTA-stabilized lutein emulsion both containing 10 mg/kg body weight of lutein at postnatal day 10. Statistically significant difference between groups at each time point was indicated by *, *P* < 0.05; **, *P* < 0.01. Mean ± SD, *n* = 3/time point/group.

The serum concentration of lutein after a one-time oral dosing was plotted versus time (Figure 3). The serum lutein concentration of the three rats that did not receive lutein was used as the baseline. Lutein was not detected in the serum and tissues of the baseline rats. At 0.5 h, the serum concentration of lutein in the free lutein group was undetectable, while the concentration in the lutein emulsion group was remarkably high (84.11 ± 23.80 vs. 0.00 ± 0.00 nmol/L, *P* < 0.01). At 4 h, the serum lutein concentration in the free lutein group reached peak value. At 6 h, the lutein emulsion group reached the peak serum lutein concentration and exhibited a significantly higher value than that of the free lutein group (308.03 ± 59.22 vs. 132.91 ± 64.96 nmol/L, *P* < 0.05). At 24 h, the serum lutein concentration in the emulsion group was significantly higher than that of the free lutein group (118.60 ± 49.16 vs. 43.16 ± 8.53 nmol/L, *P* < 0.05).

### 3.3. Acute Dosing Study—Pharmacokinetic Parameters and Bioavailability of Lutein

The key pharmacokinetic parameters of lutein in the neonatal Sprague-Dawley rats, including C_max_, AUC, and T_max_, were derived from the serum lutein kinetic curve in Figure 3 and summarized in Table 3. The lutein emulsion group showed a significantly 2.12- and 1.91- fold higher serum lutein C_max_ and AUC than that of the free lutein group, respectively (*P* < 0.05). The serum T_max_ was 6 h and 4 h in the lutein emulsion group and the free lutein group, respectively. The relative bioavailability of lutein in the emulsion as to the free lutein was 195.79%.

### 3.4. Acute Dosing Study—Tissue Lutein Profiles

**Figure 4:**
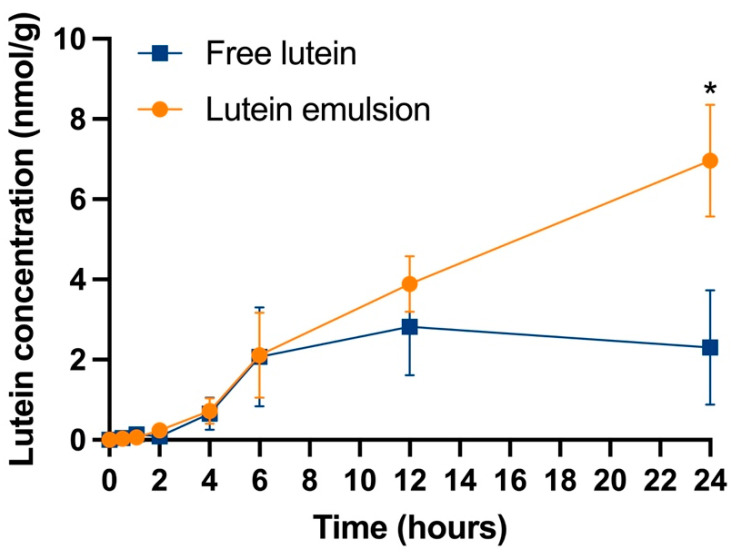
Concentration-time profile of lutein in the liver of rat pups after receiving either a single dose of free lutein or CTA-stabilized lutein emulsion both containing 10 mg/kg body weight of lutein. * indicate statistically significant difference between groups, *P* < 0.05, *n* = 3/time point/group.

The liver lutein concentration versus time points is shown in Figure 4. The liver lutein concentration in both groups continuously increased in the first 12 h. After 12 h, the liver lutein concentration in the free lutein group started to decrease, while the concentration in the lutein emulsion groups kept increasing. At 24 h, the lutein emulsion group showed a significantly higher lutein concentration than the free lutein group (6.96 ± 2.51 vs. 1.02 ± 1.18 nmol/g, *P* < 0.05).

**Figure 5:**
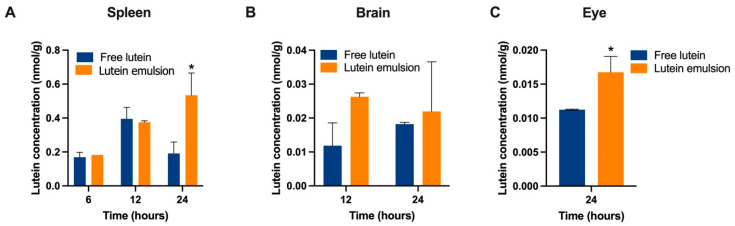
Lutein concentration in the spleen (**A**), brain (**B**), and eye (**C**) of rat pups at different time points after receiving either a single dose of free lutein or CTA-stabilized lutein emulsion both containing 10 mg/kg body weight of lutein. * indicates statistically significant difference between groups, *P* < 0.05, *n* = 3/time point/group.

The lutein concentrations in spleen, brain, and eye after a single dose are shown in Figure 5. The earliest time point for lutein to be detectable in these three organs was 6 h, 12 h, and 24 h, respectively, and therefore only these time points were included in the plot. At 24 h, a significantly higher lutein concentration was found in the spleen (0.53 ± 0.19 vs. 0.19 ± 0.10 nmol/g, *P* < 0.05) and eye (0.017 ± 0.002 vs. 0.011 ± 0.00005 nmol/g, *P* < 0.05) in the emulsion group.

### 3.5. Daily Dosing Studies—Serum and Tissue Lutein Concentrations

To assess the effects of daily consumption of CTA-stabilized lutein emulsion on the tissue lutein status in neonatal rats, two daily dosing studies were conducted. Neither study showed significant differences in body or tissue weights among groups, and daily oral lutein administration caused no observable illness in the pups (Table S1).

**Figure 6:**
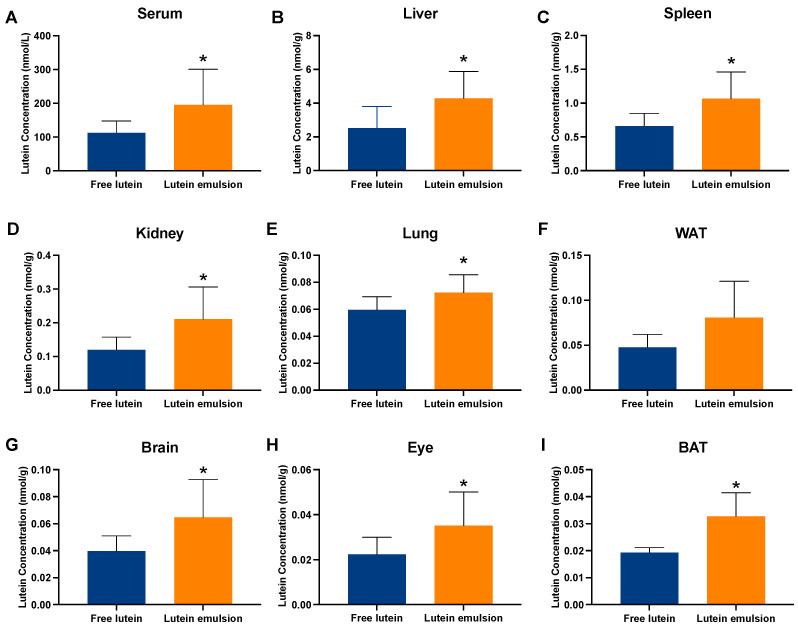
Concentration of lutein in the serum (**A**), liver (**B**), spleen (**C**), kidney (**D**), lung (**E**), white adipose tissue (WAT) (**F**), brain (**G**), eye (**H**), and brown adipose tissue (BAT) (**I**) of rat pups after receiving either free lutein or CTA-stabilized lutein emulsion both containing 2 mg/kg BW of lutein from postnatal day 5 to postnatal day 18. Rat pups were reared by mothers fed a purified diet with no lutein. * indicates statistically significant difference between groups, *P* < 0.05, *n* = 8–9/group. Graphs are ordered based on lutein concentration in the free lutein group from the highest to the lowest.

In daily dosing study 1, in which maternal rats consumed a purified diet with no lutein, after two weeks of oral dosing, rat pups in the emulsion group showed a significantly higher lutein concentration in the serum (195.76 ± 105.28 vs. 112.42 ± 34.60 nmol/L, *P* < 0.05), liver (4.29 ± 1.59 vs. 2.52 ± 1.29 nmol/g, *P* < 0.05), spleen (1.07 ± 0.39 vs. 0.66 ± 0.18, *P* < 0.05), kidney (0.21 ± 0.10 vs. 0.12 ± 0.04 nmol/g, *P* < 0.05), lung (0.07 ± 0.01 vs. 0.06 ± 0.01 nmol/g, *P* < 0.05), brain (0.06 ± 0.02 vs. 0.04 ± 0.01 nmol/g, *P* < 0.05), eye (0.04 ± 0.01 vs. 0.02 ± 0.007 nmol/g, *P* < 0.05), and BAT (0.03 ± 0.009 vs. 0.02 ± 0.002 nmol/g, *P* < 0.05), than the free lutein group (Figure 6).

**Figure 7:**
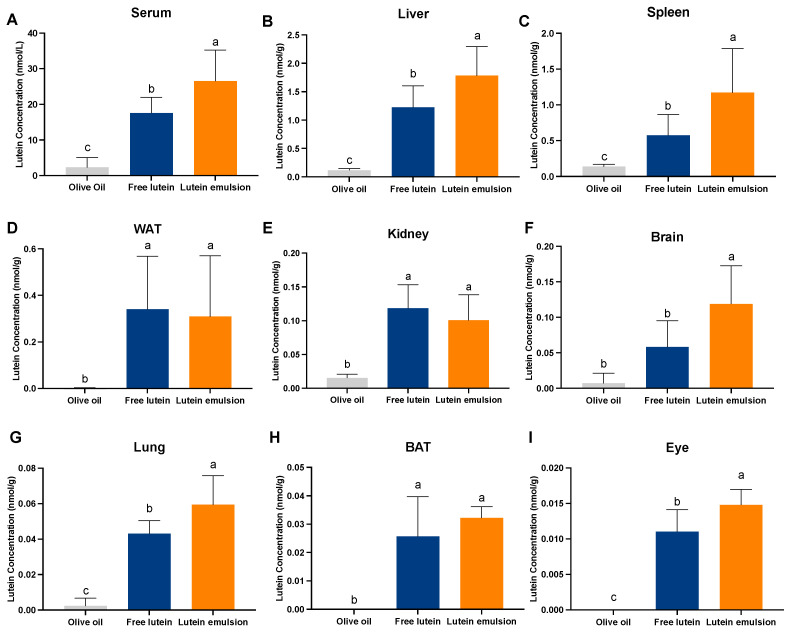
Concentration of lutein in the serum (**A**), liver (**B**), spleen (**C**), white adipose tissue (WAT) (**D**), kidney (**E**), brain (**F**), lung (**G**), brown adipose tissue (BAT) (**H**), and eye (**I**) of rat pups after receiving either free lutein or CTA-stabilized lutein emulsion both containing 2 mg/kg BW of lutein from postnatal day 5 to postnatal day 18. Rat pups were reared by mothers fed a standard chow diet containing lutein. Different letters indicate statistically significant difference among three groups, a > b > c, *P* < 0.05, *n* = 8–9/group. Graphs are ordered based on lutein concentration in the free lutein group from the highest to the lowest.

In daily dosing study 2, in which maternal rats consumed a lutein-containing standard chow diet, compared to the olive oil group, rat pups in the free lutein group had significantly higher lutein concentrations in all the analyzed organs, including the serum (17.61 ± 4.39 vs. 2.33 ± 2.77 nmol/g, *P* < 0.05), liver (1.23 ± 0.38 vs. 0.12 ± 0.03 nmol/g, *P* < 0.05), spleen (0.58 ± 0.29 vs. 0.14 ± 0.03 nmol/g, *P* < 0.05), WAT (0.34 ± 0.23 vs. 0.001 ± 0.002 nmol/g, *P* < 0.05), kidney (0.12 ± 0.03 vs. 0.016 ± 0.005 nmol/g, *P* < 0.05), brain (0.06 ± 0.04 vs. 0.007 ± 0.01 nmol/g, *P* < 0.05), lung (0.043 ± 0.007 vs. 0.002 ± 0.004 nmol/g, *P* < 0.05), BAT (0.026± 0.014 vs. 0.00 ± 0.00 nmol/g, *P* < 0.05), and eye (0.01 ± 0.003 vs. 0.00 ± 0.00 nmol/g, *P* < 0.05) (Figure 7). Compared to the free lutein group, the lutein emulsion group exhibited significantly higher lutein concentration in the serum (26.54 ± 8.74 vs. 17.61 ± 4.39 nmol/g, *P* < 0.05), liver (1.78 ± 0.51 vs. 1.23 ± 0.38 nmol/g, *P* < 0.05), spleen (1.17 ± 0.62 vs. 0.14 ± 0.03 nmol/g, *P* < 0.05), brain (0.12 ± 0.05 vs. 0.06 ± 0.04 nmol/g, *P* < 0.05), lung (0.06 ± 0.02 vs. 0.043 ± 0.007 nmol/g, *P* < 0.05), and eye (0.015 ± 0.002 vs. 0.01 ± 0.003 nmol/g, *P* < 0.05).

Comparing data of the two studies, it is interesting to note that upon receiving lutein, offspring of mothers consuming the no-lutein diet had higher lutein concentrations than those consuming the diet containing some lutein (Table 4). Specifically, the lutein concentrations in the serum, liver, and eye of the lutein emulsion group and the free lutein group were 7.37- and 6.38-, 2.41- and 2.04-, and 2.85- and 2.00-fold higher, respectively.

## 4. Discussion

To the best of the authors’ knowledge, this is the first study to evaluate the effects of an encapsulation technique on the bioavailability of lutein in a neonatal model. The findings reveal that CTA-stabilized lutein emulsion significantly enhances lutein bioavailability and its presence in key functional organs like the eye and brain.

### 4.1. Innovation and Advantages of the CTA-Stabilized Emulsion

Three delivery systems, including polymer nanoparticles, lutein nanoparticles, and lutein emulsions, are commonly used for lutein encapsulation [20]. Among these, lutein emulsion stands out as the most promising for infant food development due to its composition and texture being similar to breastmilk and its structural resemblance to milk-fat globules in breast milk. Milk-fat globules are tiny droplets of fat present in breast milk, surrounded in breast milk by a three-layer membrane comprising proteins, lipids, and carbohydrates, which act as natural emulsifiers to disperse the fat and aid in its hydrolysis and absorption by infants [48,49]. Evidence supports the concept that the unique structure of milk-fat globules might improve lutein bioavailability, potentially explaining the higher lutein levels in breastfed infants compared to those formula-fed [50]. Such similarity suggests that lutein emulsion might similarly enhance lutein absorption in infants.

In this study, the food-grade biopolymer CTA was utilized as an emulsifier to develop lutein emulsions. This emulsifier offers advantages over synthetic emulsifiers, which is not as label friendly for infant food preparation [51]. Biopolymers, including proteins like soy protein and casein, and polysaccharides such as starch and carboxymethyl cellulose, are not only food-grade colloids but also function as effective stabilizers in O/W emulsions [52]. Among these, plant-derived polysaccharides, particularly starch, are often preferred to being used. Their advantages include the absence of food allergy concerns when used in infant products, along with their cost-effectiveness due to low price and wide availability. Furthermore, native starches can be chemically modified to enhance their functionality. For instance, OS starches are a type of modified starch. These starches are produced by reacting corn, potato, or other starches with octenylsuccinic anhydride, which adds carboxylate groups to the starch molecules. The resulting water-soluble starch, possessing both hydrophilic and lipophilic properties, is an effective emulsifier for O/W emulsions as compared to native starch [51].

### 4.2. Characteristics of the CTA-Stabilized Lutein Emulsion

The CTA-stabilized lutein emulsion showed homogenized droplets according to the confocal images. CTA at the oil/water interface resulted in the formation of a dense layer surrounding the surface of the spherical oil droplets, preventing droplet coalescence and potentially enhancing the stability of the emulsion. This observation also corresponds to the visual appearance of the gel-like emulsion. Emulsion gels are structurally stable under long-term storage and could serve as delivery carriers for lipophilic bioactive compounds [31]. The mean droplet size of the emulsion, which is 1.73 ± 1.14 μm, was within the range of conventional emulsion (i.e., 100 nm to 100 μm) [53]. This relatively small droplet size is crucial for lutein bioavailability, as it increases the surface area available for gastrointestinal interactions [24].

### 4.3. CTA-Stabilized Lutein Emulsion Enhanced Lutein Bioavailability in Neonatal Rats

AUC_0–24_, which is the area under the plot of serum concentration of the biocompound versus time after a single dosing, reflects the bioavailability of the compound [54]. In the acute dosing study, it was found that the relative bioavailability of lutein from the emulsion as to free lutein was 195.57%, calculated as the ratio of AUC_0–24_ of the emulsion group to the free lutein group, indicating an almost two-fold higher bioavailability. This aligns with a previous study where oral fast-dissolving lutein nanocrystals achieved a relative bioavailability of 207.67% [37]. The serum T_max_, or time to serum peak concentration, was 4 h for free lutein and 6 h for the emulsion group, which were both within the previously reported range of 0.25 to 6 h (Table 5). Polymer, nanoparticles, and emulsion are the main lutein delivery systems [20], with nanoparticles typically shortening T_max_. However, in this study, the T_max_ of the lutein emulsion group was 2 h later than the free lutein group. Sato et al. found a similar delay in T_max_ for lutein in an emulsion gel compared to a powder-like encapsulated lutein (Table 5) [55]. This delay may be due to the polysaccharides in the gel state, which can prolong gastric emptying, increasing retention in the stomach and small intestine compared to free lutein [37]. Gelation of the emulsion might also delay the diffusion of digestive enzymes, controlling the release of lutein [56].

It was noted that serum lutein concentration in the emulsion group rapidly increased and then decreased 0.5 h post administration (Figure 2), a pattern also observed in other studies with encapsulated lutein [36,55]. It could be due to different absorption mechanisms between free lutein and lutein emulsion. Lutein forms mixed micelles in the small intestine, is absorbed via passive diffusion, packed into chylomicrons, and secreted into the mesenteric lymph stream, and then follows chylomicron metabolism, eventually entering the bloodstream in lipoproteins [60,61]. Free lutein consumption, under normal conditions, results in a gradual increase in the serum level, as seen in the free lutein group. In contrast, the emulsion may alter lutein absorption; its water solubility could increase, enhancing transport across intestinal epithelial cells and directly to the liver via the hepatic portal vein [60,62]. This could explain the initial small peak in blood lutein concentration at 0.5 h in the emulsion group. Subsequently, lutein stored in the liver regulates serum concentrations, normalizing levels over time.

Lutein concentration in the liver, spleen, and eye were found to be significantly higher in the lutein emulsion group than that of the free lutein group at 24 h. These results align with the findings of Sato et al., Arunkumar et al., and Wu et al., who observed increased lutein concentrations in the liver and/or eyes with lutein delivery systems in mouse models 4 to 24 h after a single dosing [54,55,63].

### 4.4. CTA-Stabilized Lutein Emulsion Improved Tissue Lutein Status

A daily feeding or dosing study would provide insight into the tissue distribution or physiologic stores of a bio-compound. We conducted two daily dosing studies, in which maternal rats consumed a no-lutein or a lutein-containing diet. With the maternal diet containing no lutein, it would eliminate the potential impact of dietary lutein on the effect of the lutein emulsion. With the lutein-containing diet, it would more mimic human dietary lutein consumption. It was found that, regardless of the maternal diet, lutein emulsion significantly increased the lutein concentration in many tissues, including the eye and the brain.

As a lipophilic bio-compound, lutein accumulates in fatty tissues, i.e., the liver and WAT, for long-term storage. Once taken up by the liver, lutein can either be stored or redistributed to peripheral tissues via very low-density lipoproteins [64]. The current study showed that liver had the highest lutein concentrations among all organs in both free lutein and lutein emulsion groups, but the emulsion group had 1.44 to 1.91-fold higher concentrations than the free lutein group. Elevated liver lutein concentration may suggest a greater availability of lutein for utilization in target tissues when required [12]. Interestingly, in both studies, no significant difference was found in WAT lutein concentration between the free lutein and lutein emulsion group. Similar findings were reported by Murillo et al. in Guinea pigs [65]. WAT lutein concentration, a long-term dietary lutein indicator, was suggested to have a negative correlation with macular pigment optical density (MPOD) in adults, indicating a competitive relationship between adipose tissue and the retina for serum lutein [66]. Studies indicated that individuals with higher body fat or obesity had lower MPOD [67,68], implying that WAT might sequester lutein, reducing its availability to target organs. Notably, our results suggest that lutein emulsion selectively enhances lutein delivery to functional organs without increasing WAT storage in neonatal rats [46].

It was exciting to find that the two major target organs of lutein, i.e., the eye and brain, exhibited higher lutein concentration in the lutein emulsion group. Lutein and zeaxanthin are the only dietary carotenoids that cross the blood-retina barrier [15]. They act as antioxidants, anti-inflammatory agents, and the main component of macular pigment in the human eye. Jeon et al. found that lutein predominantly accumulates in the occipital cortex, the region of the human brain identified as the primary visual cortex, which plays a key role in processing visual information [69]. Increased lutein concentrations in the eye and brain may benefit visual and cognitive developments during infancy, particularly for at-risk infants.

Furthermore, the lutein emulsion group showed significantly higher lutein concentration in the lung. Human lung development, critical from embryonic stages through early childhood, is susceptible to changes due to early oxidative stress exposure. This is particularly relevant for preterm infants prone to neonatal respiratory conditions [70]. Studies in adults have shown lutein’s positive impact on lung function, with correlations observed between lutein levels and reduced severity of emphysema and improved lung function in smokers [71,72]. Lutein supplementation has also been noted to decrease lung inflammation and oxidative stress in pollution-exposed mice [73]. The potential role of lutein in lung health might be linked to its interaction with retinoic acid receptors (RARs), crucial in alveolar development [74]. Our findings suggest the potential benefit of lutein emulsion in early lung development, but further research is required to fully understand the roles of lutein in lung development in neonates.

A high lutein concentration was noted in the neonatal spleen, which possessed the second highest concentration among all organs, and lutein emulsion further increased the concentration. Studies in adult rodents and infant nonhuman primates also show substantial spleen accumulation of lutein [54,69,75]. For instance, Jeon et al. found the spleen to have the third-highest lutein concentration in infant Rhesus Macaques, after the liver and retina [69]. The roles of spleen in neonates include immune system regulation, red blood cell production, and cell removal [76]. Given that infants, particularly preterm ones, have immature immune systems and are vulnerable to oxidative stress, the effectiveness of lutein in immune stimulation and tumor growth inhibition in mice is noteworthy [77,78]. Lutein supplementation has shown dose-dependent enhancements in adult mice’s immune functions, such as increased natural killer cell activity and lymphoproliferative response in the spleen [79]. While the specific functions of lutein in the neonatal spleen remain unclear, it may aid spleen development and immunomodulation [80,81]. In this study, the lutein emulsion group displayed higher spleen lutein concentration, potentially benefiting spleen development and neonatal rat immunity.

### 4.5. Strengths and Limitations of the Study

The current study had several advantages. First, we utilized a food-grade biopolymer, OS starch, which is an approved food additive in infant foods, as the emulsifier [32]. Previous reports suggested that there is no safety concerns for the usage of OS starch within the range reported in clinical studies [32,82,83]. In this study, we used OS starch at levels (based on the body weight of rat pups) well below those reported in clinical studies. Secondly, the study encompassed both acute and daily dosing evaluations to assess the effects of lutein emulsion. Thirdly, the 2 mg/kg body weight dosage employed in the daily dosing studies, derived from human infant studies and established as an Acceptable Daily Intake for lutein, enhances the translational relevance of the current research to human clinical trials [43,44,45].

While the Sprague-Dawley rat is a commonly employed model for investigating lutein metabolism and function, it is important to recognize that there are notable differences in lutein metabolism between rodents and humans. This discrepancy is attributed to the high activity of the xanthophyll cleavage enzyme beta-carotene oxygenase 2 (BCO2) in rodents, which is inactive in humans. Consequently, rodents experience much lower lutein accumulation in their eyes [84]. To better mimic human lutein metabolism, a BCO2 knockout (BCO2^-/-^) mouse model has emerged in recent years [85,86].

Nevertheless, the data obtained from the current model remains valuable, clearly demonstrating the efficacy of CTA-stabilized lutein emulsion in enhancing lutein bioavailability. In our future studies, we will use the BCO2^-/-^ model to further refine our understanding of the in vivo effect of the lutein emulsion in a system that more closely mimics human physiology.

## 5. Conclusions and Future Directions

A biopolymer-stabilized emulsion was shown to be an efficient and safe system for enhancing the bioavailability of lutein in a neonatal rat model. This emulsion system holds promise as a cost-effective fortification in infant formula and other baby foods, optimizing the bioavailability of lutein and thereby promoting early-life health and development. Furthermore, future research should investigate the impact of the lutein emulsion in diverse neonatal disease models, including conditions such as retinopathy of prematurity.

## Figures and Tables

**Table 1:** Diet composition of the purified diet with no lutein. Formulation details are provided in grams, g, and kilocalories, kcal.

Purified Diet ^1^		
Macronutrients	g% ^2^	kcal%
Fat	12	25
Carbohydrate	57	55
Protein	21	20
Total	-	100
kcal/g	4.2	-
Ingredient	g	kcal
**Lutein**	**0**	**0**
Casein	200	800
L-Cystine	3	12
Corn Starch	353.8	1415
Maltodextrin 10	125	500
Sucrose	68.8	275
Cellulose, BW200	50	0
Soybean Oil	25	225
Lard	87.7	789
Mineral Mix S10026	10	0
DiCalcium Phosphate	13	0
Calcium Carbonate	5.5	0
Potassium Citrate, 1 H_2_O	16.5	0
Vitamin Mix V10001	10	40
Choline Bitartrate	2	0
Food Color	0.05	0

^1^ Research Diets, Inc (D19120501), purified diet with no lutein. ^2^ The number of grams of the nutrient is given by per 100 g of the total diet.

**Table 2:** Mean, minimum, and maximum droplet diameter of the lutein emulsion stabilized by 30% *w*/*v* capsule TA^®^ (CTA).

Mean Droplet Diameter ± SD (μm)	Minimum Droplet Diameter (μm)	Maximum Droplet Diameter (μm)	Encapsulation Efficiency (%)
1.73 ± 1.14	0.08	4.99	0.89 ± 0.014

**Table 3:** Pharmacokinetic parameters of lutein in rat pups after received either a single dose of free lutein or CTA-stabilized lutein emulsion both containing 10 mg/kg body weight of lutein (*n* = 3/groups/time).

Parameters	Lutein Emulsion	Free Lutein
C_max_ (nmol/L) ^1^	308.03 ± 59.22 *	145.00 ± 49.00
T_max_ (h)	6	4
AUC_0–24_ (nmol/L∗h)	3804 ± 606.50 *	1987 ± 247.90
Relative bioavailability% ^2^	195.79	

^1^ C_max_, maximum lutein concentration; T_max_, time to reach C_max_; AUC_0–24_, area under the concentration-time curve to terminal time. * indicates statistically significant difference between groups (*P* < 0.05). ^2^ Relative bioavailability (%) = $\frac{AUCexperiment}{AUCcontrol}\times 100$.

**Table 4:** Lutein concentration in the serum and tissues of rat pups reared by mothers fed with a purified diet with no lutein and a standard chow diet containing lutein.

	Purified Diet with No Lutein		Standard Chow Diet Containing Lutein		
Lutein Concentration (nmol/L) or (nmol/g)	Free Lutein Group	Lutein Emulsion Group	Olive Oil Group	Free Lutein Group	Lutein Emulsion Group
Serum	112.42 ± 34.60 ^a^	195.76 ± 105.28 ^a^	2.33 ± 2.77 ^c^	17.61 ± 4.39 ^b^	26.54 ± 8.74 ^b^
Liver	2.52 ± 1.29 ^b^	4.29 ± 1.59 ^a^	0.12 ± 0.03 ^c^	1.23 ± 0.38 ^bc^	1.78 ± 0.51 ^b^
Eye	0.04 ± 0.01 ^a^	0.02 ± 0.007 ^a^	Below LOD *	0.01 ± 0.003 ^c^	0.015 ± 0.002 ^b^
Spleen	0.66 ± 0.18 ^a^	1.07 ± 0.39 ^a^	0.14 ± 0.03 ^c^	0.58 ± 0.29 ^b^	1.17 ± 0.62 ^a^
Kidney	0.12± 0.04 ^b^	0.21 ± 0.10 ^a^	0.016 ± 0.005 ^c^	0.12 ± 0.03 ^b^	0.10 ± 0.04 ^b^
Lung	0.06 ± 0.01 ^a^	0.07 ± 0.01 ^a^	0.002 ± 0.004 ^b^	0.043 ± 0.007 ^a^	0.06 ± 0.02 ^a^
Brain	0.04 ± 0.01 ^b^	0.06 ± 0.02 ^a^	0.007 ± 0.01 ^b^	0.06 ± 0.04 ^ab^	0.12 ± 0.05 ^a^
WAT	0.08 ± 0.04 ^a^	0.05 ± 0.01 ^a^	0.001 ± 0.002 ^b^	0.34 ± 0.23 ^a^	0.31 ± 0.26 ^a^
BAT	0.02 ± 0.002 ^a^	0.03 ± 0.009 ^a^	Below LOD	0.026 ± 0.014 ^a^	0.03 ± 0.004 ^a^

* LOD, limit of detection. WAT, white adipose tissue; BAT, brown adipose tissue. Data represent Mean ± SD, different letters indicate statistically significant difference among five groups in each organ, a > b > c, *P* < 0.05.

**Table 5:** Summary of earlier studies reporting the serum T_max_ of free lutein and lutein in different encapsulation systems.

			T_max_ (h)	
Author (Year of Publication)	Encapsulation Techniques	Animal Model	Encapsulated Lutein	Free Lutein
Arunkumar et al. (2015) [42]	Polymer	Mice	4	4
Ranganathan et al. (2019) [57]	Polymer	Mice	4	4
Zhang et al. (2015) [58]	Nanoparticles	Sprague-Dawley rats	4.7 ± 3.0	6 ± 2.2
Wu et al. (2019) [54]	Nanoparticles	Sprague-Dawley rats	0.25	2
Liu et al. (2017) [37]	Nanoparticles	Sprague-Dawley rats	0.25	2
Chang et al. (2018) [59]	Nanoparticles	Sprague-Dawley rats	3	3
Sato et al. (2018) [55]	Nanoparticle	Wistar rats	2	NA
Sato et al. (2018) [55]	Emulsion	Wistar rats	4	NA

NA, not available.

## Data Availability

The raw data supporting the conclusions of this article will be made available by the authors on request.

## Supplementary Materials

The following supporting information can be downloaded at: https://www.mdpi.com/article/10.3390/nu16030422/s1, Figure S1: A representative UPLC chromatogram of lutein in the liver of a rat pup 24 h after a single dose of lutein emulsion. Figure S2: Diet composition of the standard chow diet. Table S1: Body weight and tissue weights of rat pups among groups at postnatal day 19.
