# Supplementary material for: Utilization of Biopolymer-Based Lutein Emulsion as an Effective Delivery System to Improve Lutein Bioavailability in Neonatal Rats
Source: Nutrients. 2024 Jan 31;16(3):422. doi: 10.3390/nu16030422 (PMC10857328; doi:10.3390/nu16030422)
Supplement: Supplementary file 1 [file nutrients-16-00422-s001.zip › nutrients-2823406-supplementary.pdf]

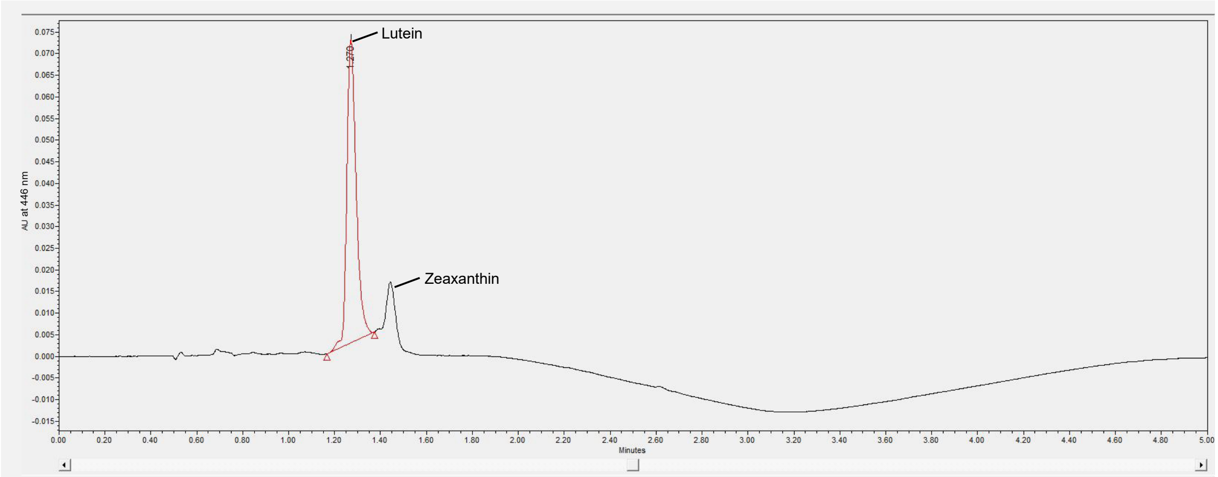

Figure S1. A representative UPLC chromatogram of lutein in the liver of a rat pup 24 hours after a single dose of lutein emulsion.

7012

ENVIGO

Teklad LM-485 Mouse/Rat Sterilizable Diet

Product Description- 7012 is a fixed formula, autoclavable diet manufactured with high quality ingredients and designed to support growth and reproduction of rodents. Typical isoflavone concentrations (daidzein + genistein aglycone equivalents) range from 300 to 600 mg/kg. Absence of animal protein and fish meal minimizes the presence of nitrosamines. 7012 is supplemented with additional vitamins to ensure nutritional adequacy after autoclaving. **Also available certified (7012C) and irradiated (7912).**

Ingredients (in descending order of inclusion)- Ground corn, dehulled soybean meal, ground oats, wheat middlings, dehydrated alfalfa meal, soybean oil, corn gluten meal, calcium carbonate, dicalcium phosphate, brewers dried yeast, iodized salt, choline chloride, kaolin, magnesium oxide, L-lysine, DL-methionine, ferrous sulfate, menadione sodium bisulfite complex (source of vitamin K activity), vitamin E acetate, thiamin mononitrate, calcium pantothenate, manganous oxide, niacin, copper sulfate, zinc oxide, vitamin A acetate, pyridoxine hydrochloride, riboflavin, vitamin D_3 supplement, vitamin B_{12} supplement, folic acid, biotin, calcium iodate, cobalt carbonate.

Standard Product Form: Pellet

**Macronutrients**		
Crude Protein	%	19.1
Fat (ether extract) [a]	%	5.8
Carbohydrate (available) [b]	%	44.3
Crude Fiber	%	4.6
Neutral Detergent Fiber [c]	%	13.7
Ash	%	6.1
Energy Density [d]	kcal/g (kJ/g)	3.1 (13.0)
Calories from Protein	%	25
Calories from Fat	%	17
Calories from Carbohydrate	%	58
**Minerals**		
Calcium	%	1.0
Phosphorus	%	0.7
Non-Phytate Phosphorus	%	0.4
Sodium	%	0.3
Potassium	%	0.8
Chloride	%	0.5
Magnesium	%	0.2
Zinc	mg/kg	63
Manganese	mg/kg	93
Copper	mg/kg	23
Iodine	mg/kg	3
Iron	mg/kg	240
Selenium	mg/kg	0.16
**Amino Acids**		
Aspartic Acid	%	1.8
Glutamic Acid	%	2.8
Alanine	%	1.0
Glycine	%	0.8
Threonine	%	0.8
Proline	%	1.4
Serine	%	1.3
Leucine	%	1.7
Isoleucine	%	0.8
Valine	%	0.9
Phenylalanine	%	0.9
Tyrosine	%	0.8
Methionine	%	0.4
Cystine	%	0.3
Lysine	%	1.0
Histidine	%	0.5
Arginine	%	1.2
Tryptophan	%	0.3

**Vitamins**		
Vitamin A [e, f]	IU/g	30.0
Vitamin D_3 [e, g]	IU/g	2.4
Vitamin E	IU/kg	150
Vitamin K_3 (menadione)	mg/kg	80
Vitamin B_1 (thiamin)	mg/kg	95
Vitamin B_2 (riboflavin)	mg/kg	14
Niacin (nicotinic acid)	mg/kg	100
Vitamin B_6 (pyridoxine)	mg/kg	17
Pantothenic Acid	mg/kg	87
Vitamin B_{12} (cyanocobalamin)	mg/kg	0.09
Biotin	mg/kg	0.77
Folate	mg/kg	7
Choline	mg/kg	2200
**Fatty Acids**		
C16:0 Palmitic	%	0.6
C18:0 Stearic	%	0.2
C18:1ω9 Oleic	%	1.3
C18:2ω6 Linoleic	%	2.6
C18:3ω3 Linolenic	%	0.3
Total Saturated	%	0.8
Total Monounsaturated	%	1.3
Total Polyunsaturated	%	2.9
**Other**		
Cholesterol	mg/kg	--

[a] Ether extract is used to measure fat in pelleted diets, while an acid hydrolysis method is required to recover fat in extruded diets. Compared to ether extract, the fat value for acid hydrolysis will be approximately 1% point higher.

[b] Carbohydrate (available) is calculated by subtracting neutral detergent fiber from total carbohydrates.

[c] Neutral detergent fiber is an estimate of insoluble fiber, including cellulose, hemicellulose, and lignin. Crude fiber methodology underestimates total fiber.

[d] Energy density is a calculated estimate of *metabolizable energy* based on the Atwater factors assigning 4 kcal/g to protein, 9 kcal/g to fat, and 4 kcal/g to available carbohydrate.

[e] Indicates added amount but does not account for contribution from other ingredients.

[f] 1 IU vitamin A = 0.3 μg retinol

[g] 1 IU vitamin D = 25 ng cholecalciferol

For nutrients not listed, insufficient data is available to quantify.

Nutrient data represent the best information available, calculated from published values and direct analytical testing of raw materials and finished product. Nutrient values may vary due to the natural variations in the ingredients, analysis, and effects of processing.

Teklad Diets are designed and manufactured for research purposes only.

SGS

© 2015 Envigo

0915

Teklad Diets + Madison WI + envigo.com + tekladinfo@envigo.com + (800) 483-5523

Figure S2. Diet composition of the standard chow diet.

Table S1. Body weight and tissue weights of rat pups among groups at postnatal day 19[*]

	Purified diet with no lutein		Standard chow diet containing lutein		
	Free lutein group	Lutein emulsion group	Olive oil group	Free lutein group	Lutein emulsion group
Body weight, g	39.89 ± 0.37[a]	41.23 ± 0.96[a]	39.26 ± 0.80[a]	38.52 ± 0.86[a]	37.72 ± 0.80[a]
Liver, g	1.54 ± 0.06[b]	1.46 ± 0.03[a]	1.25 ± 0.06[a]	1.56 ± 0.09[a]	1.56 ± 0.04[a]
Eye, g	0.12 ± 0.01[a]	0.12 ± 0.005[a]	0.12 ± 0.003[a]	0.11 ± 0.003[a]	0.11 ± 0.006[a]
Lung, g	0.56 ± 0.04[a]	0.053 ± 0.07[a]	0.53 ± 0.02[a]	0.53 ± 0.02[a]	0.55 ± 0.02[a]
Kidney, g	0.46 ± 0.02[b]	0.42 ± 0.04[a]	0.41 ± 0.03[a]	0.38 ± 0.01[a]	0.38 ± 0.005[a]
WAT, g	0.22 ± 0.02[a]	0.26 ± 0.03[a]	0.20 ± 0.03[a]	0.24 ± 0.02[a]	0.28 ± 0.02[a]
Brain, g	1.24 ± 0.004[b]	1.27 ± 0.05[a]	1.17 ± 0.02[a]	1.23 ± 0.04[a]	1.25 ± 0.03[a]
Spleen, g	0.16 ± 0.02[a]	0.15 ± 0.02[a]	0.14 ± 0.006[a]	0.14± 0.003[a]	0.13 ± 0.004[a]
BAT, g	0.15 ± 0.002[a]	0.15 ± 0.04[a]	0.13 ± 0.02 [a]	0.10 ± 0.01[a]	0.16 ± 0.009[a]

[*]Values represent Mean ± SD, *n* = 8-9 pups/group. Different letters indicate statistically significant difference among five groups, a > b > c, P < 0.05. WAT, white adipose tissue; BAT, brown adipose tissue.
